# Editors’ Book Table

**Published:** 1872-06

**Authors:** 


### Editors' Book Table.

[NOTE.—All works reviewed in the columns of the CHICAGO MEDICAL JOURNAL may be found in the extensive stock of W. B. KEEN, COOKE & CO., whose catalogue of Medical Books will be sent to any address upon request.]

### BOOKS RECEIVED.

*Catalogue of the Library of the Surgeon-General's Office, United States Army.* With an Alphabetical Index of Subjects. Washington: Government Printing Office. 1872. 4to, pp. 454. Also, Supplement to Catalogue No. 1. List of American Medical Journals. Pp. 27.

*Transactions of the Twenty-first Anniversary Meeting of the Illinois State Medical Society.* Held at Peoria, May 16, 1871. Chicago: Fergus Printing Company. 1872. Pp. 111.

The "Transactions" contain Dr. D. Prince's Report on Plastic and Orthopedic Surgery, "presented by the Author to his Friends and Eminent Members of the Profession as a token of respect." This Report is also furnished by Messrs. Lindsay & Blakiston, bound in connection with the two former reports.

*Insanity and Insane Asylums.* Report of E. T. Wilkins, M.D., Commissioner in Lunacy for the State of California. Made to His Excellency H. H. Haight, Governor, Dec. 2, 1871. Sacramento: T. A. Springer, State Printer. 1872. Pp. 345.

To the whole is appended quite a number of large diagrams of buildings, etc., etc.

PAMPHLETS.

*The Sun and the Phenomena of its Atmosphere.* By Prof. C. A. YOUNG, Ph.D., of Dartmouth College. New Haven, Conn.: Charles C. Chatfield & Co. 1872. Price, 25c.

This is No. 8 of the University Series, to which we have often directed our readers' attention. It fully sustains the reputation achieved by its predecessors. It is announced as the intention to publish ten numbers each year, and, hereafter, especial preference is to be given to lectures and essays, by American scientists, which embody the results of original research and study. Each number, singly, 25c; five successive numbers, in advance, $1.10; ten successive numbers, $2.00. The First Series (five numbers), with a general introduction by Noah Porter, D.D., etc., President Yale College, bound, mailed at $1.50.

*Inaugural Addresses at Washington University, St. Louis*, Feb. 29, 1872. With an Appendix.

*The Limit of the Police Power in the Control of Corporations.* A Statement of the Condition, Property and Franchises of the Graceland Cemetery Co.; together with a Review of the Legislation concerning Cemeteries in Lake View. By WM. C. REYNOLDS.

This is a very important paper, being general in its discussion of the subject, although elicited by an attempt on the part of certain township authorities to void certain alleged vested rights of a particular Cemetery Co.

*Second Annual Announcement of the St. Paul School for Medical Instruction.* 1872.

We reprint the closing portion of the circular:

The course of study includes recitations and lectures upon Surgery, Therapeutics, Materia Medica, Diseases of Children, Anatomy, Chemistry, Physiology, Pathology, Medical Jurisprudence, Principles and Practice of Medicine, Obstetrics and Diseases of Women.

The text-books used are Gross and Erichsen's Surgery, Gray's Anatomy, Wells' Chemistry, Stille's Therapeutics and Materia Medica, or the Dispensatory, Meigs and Pepper on Diseases of Children, Dalton's Physiology, Flint's or Wood's Practice of Medicine, Bedford's Obstetrics, Thomas on Diseases of Women, and Sim's Uterine Surgery.

The summer term for 1872 will commence on Thursday, June 6th, and continue 16 weeks.

| | |
|---|---|
| The fees for the whole year (excluding dissections) are | $50 00 |
| For the summer term alone | 30 00 |
| For the winter term alone (excluding dissections)* | 25 00 |

Good board can be had in the city for from $4.00 to $10.00 per week.

Arrangements can be made so that a limited number of students who are unable to pay their way can obtain their tickets on favorable terms.

OBJECT.

The object of this school is not to represent, or in any way take the place of a regular *College*, but to prepare students for a better understanding of the lectures which they will hear in the College course, and to drill them more thoroughly in the elementary branches than can be done in the short time allowed by Colleges for instruction, and the arrangement of terms of study will be such that they will not interfere with the winter course of the Chicago College.

Students on arriving in St. Paul will call at once on the Secretary, who will assist them in finding board, etc.

For any further particulars, address ALEX. J. STONE, M.D., *Secretary*, No. 34 Jackson Street, St. Paul, Minn.

* Dissection tickets, $10.00 each, to be had at option of Dr. Richeson.

